# Screening the antimicrobial properties of snake venoms: an iterative fractionation approach

**DOI:** 10.1099/acmi.0.001151.v3

**Published:** 2026-09-02

**Authors:** Alice J. Fraser, Mark C. Wilkinson, Conor J. Meehan, Nicholas R. Casewell, Adam P. Roberts, Alasdair T.M. Hubbard

**Affiliations:** 1 Department of Tropical Disease Biology, Liverpool School of Tropical Medicine, Pembroke Place, Liverpool L3 5QA, UK; 2 Centre for Drugs and Diagnostics, Liverpool School of Tropical Medicine, Pembroke Place, Liverpool L3 5QA, UK; 3 Centre for Snakebite Research and Interventions, Liverpool School of Tropical Medicine, Pembroke Place, Liverpool L3 5QA, UK; 4 Department of Biosciences, Nottingham Trent University, Clifton Campus, College Drive, Clifton, Nottingham, NG11 8NS, UK; 5 School of Veterinary Medicine and Sciences, University of Nottingham, Sutton Bonington Campus, Sutton Bonington, Leicestershire, LE12 5RD, UK

**Keywords:** antibiotic discovery, antimicrobials, *Escherichia coli*, phospholipase A₂, snake venom

## Abstract

Antimicrobial resistance increasingly poses a significant global health challenge. New antimicrobials are desperately needed, driving the need to explore novel sources of natural products. Snake venom has shown promise due to the complex mixture of proteins and peptides which demonstrate therapeutic utility in other aspects of healthcare. In this study, we took an iterative approach to screen 14 snake venoms against *Escherichia coli* and *Klebsiella pneumoniae* and identify venom-derived proteins with diverse activity against *E. coli*. Six venoms exhibited significant near-complete inhibitory activity, and four were retained after dialysis, confirming that the inhibitory effect was due to high-molecular-weight components above 3.5 kDa. Fractionation of two active venoms from *Naja mossambica* and *Naja subfulva* revealed protein fractions which have either subtle or significant antimicrobial activity. SDS-PAGE and mass spectrometry identified phospholipase A₂ isoforms in *N. mossambica* venom fractions and neurotoxic proteins (long and short neurotoxins) in *N. subfulva* venom fractions as potential antimicrobials.

## Data Summary

All raw data generated in this project and code for graphs generated in R and statistical analysis are freely available here: https://github.com/Alhubb/Antimicrobial-proteins-in-snake-venom.

## Introduction

The antimicrobial resistance (AMR) crisis is continuing to escalate, with 1.14 million global deaths directly attributed to AMR in 2021 alone and predicted to rise to 8.22 million per year by 2025 [1]. AMR threatens the prevention and treatment of infections caused by a range of bacterial pathogens. Discovery and development of new antibiotics, especially those exhibiting novel mechanisms of action, are crucial to combat antimicrobial-resistant infections, particularly those caused by *Enterobacteriaceae* [1–4]. For this reason, there has been increasing interest in underexploited sources of pharmacologically active macromolecules. Venom from diverse animals, including spiders, honeybees, ants, wasps and scorpions, has been investigated for their antimicrobial activity, computationally and experimentally, identifying a wide range of proteins [5, 6]. Due to its diverse protein and peptide composition, snake venom may be a valuable resource for drug discovery [7–9]. Furthermore, as the therapeutic potential of snake venom has previously been demonstrated for hypertension [6, 10, 11] and other cardiac conditions [6, 12], there is precedent to explore the potential of venom-derived proteins as pharmaceuticals, including antimicrobials [5, 6, 13].

Studies into the antimicrobial properties of snake venom have primarily focused on venom from snakes of the Elapidae (elapids) and Viperidae (vipers) families, and variation in antimicrobial efficacy has been attributed to differences in venom composition [14]. Elapid and viper venom is composed of up to 59 known protein families, but it is estimated that many more proteins and peptides may be present but currently unidentified [15]. Furthermore, snake venom composition is highly variable, even within snakes of the same species, and can be altered by a host of external and physiological factors, including diet [16], sex [17] and geographical location [18]. This provides the opportunity to screen and identify novel proteins from even a single species. Despite this protein diversity, 90% of elapid and viper venom is restricted to 8 and 11 protein families, respectively. The dominant protein families include phospholipase A_2_s (PLA_2_s), metalloproteases, serine proteases and (primarily restricted to elapids) three-finger toxins (3FTxs). Unsurprisingly, as these proteins are well characterized due to their abundance and toxicity, most studies into venom-derived antimicrobial activity have been focused on these major constituents, with antimicrobial activity attributed to disruption of the bacterial membrane. Collective evidence from independent studies has highlighted that there are numerous mechanisms by which this is achieved [7, 19–22], dictated by the biochemical characteristics of the protein which may vary depending on isoform, which is intrinsically linked to snake species [15].

Other minor protein and peptide components of snake venom have also been investigated, and their biological activities and structure warrant further investigation for antimicrobial activity. For example, l-amino acid oxidases catalyse the production of reactive oxygen species, which are antimicrobial to a range of pathogens [23], and the WAP domain of the snake venom protein omwaprin [24] is found in a class of antimicrobial proteins that are also mediators of the innate immune system [25]. Previously characterized antimicrobial peptides are also found in snake venom, such as cathelicidins, which are effector molecules of the innate immune system and have both antibacterial and antifungal effects [26, 27].

Undertaking broad screening of crude snake venoms has proved a promising strategy, particularly in the search for antimicrobial proteins. However, in most cases, investigations are restricted to determining bacterial susceptibility by clinical methods [28–32], which, other than indicating which venoms should be investigated further, has limited research utility. An alternative approach has been to directly test the dominant proteins of snake venoms against bacteria, as their proteolytic activity and ability to disrupt mammalian cell homeostasis [33] have led researchers to hypothesize that bacterial cells may also be subject to the same effects. However, this process may lead to bias towards known and major classes of proteins and ignore less represented proteins which have antimicrobial activity. In a small number of studies, further fractionation of venom has been undertaken, enabling the isolation and downstream identification of specific proteins [34, 35]. Again, these studies employ clinical methods to determine antimicrobial activity, such as disc diffusion [35], which may lead to missing subtle, but significant, inhibition of growth. This presents a barrier for the discovery of minor venom components, which may be a valuable source of non-toxic [36], pharmacologically active molecules [15], and a suitable starting point for the development of novel antibiotics.

In this study, we developed an assay to assess the antimicrobial activity of whole venom from 14 species of snake against *Escherichia coli* and *Klebsiella pneumoniae* and then employed an iterative approach to identify the protein fractions of two active whole venoms from *Naja mossambica* and *Naja subfulva*. From these protein fractions, we were able to determine the protein classes present in the sample. The ability to identify and profile particularly active fractions demonstrating antimicrobial activity will enable us to further focus efforts in future screening rounds and to further purify and determine the exact protein(s) responsible for antimicrobial activity and ascertain the mode of action.

## Methods

### Bacterial strains, media and antibiotics

*E. coli* NCTC 86 and *K. pneumoniae* NCTC 13696, sourced from the National Collection of Type Cultures, were grown on Luria–Bertani (LB) agar (Sigma, UK) at 37° for 18 h. Single colonies were selected and sub-cultured into 10 ml of cation-adjusted Mueller-Hinton broth (CA-MHB) (Sigma, UK) and grown for 18 h at 37 °C, 200 r.p.m. Ciprofloxacin (Sigma, UK) was solubilized to a concentration of 1 mg ml^−1^ in 0.1 N hydrochloric acid (Sigma, UK) and filter-sterilized through a 0.22 µm polyethersulfone filter unit (Millipore, USA).

### Snake venom

Whole venom from *Daboia russelii* from Sri Lanka, *Naja nigricollis* from Nigeria (NGA) and Tanzania (TZA), *Calloselasma rhodostoma* (captive bred), *Bitis arietans*, *Naja naja* (captive bred), *Dendroaspis polylepis* from TZA, *N. mossambica* from TZA, *Naja pallida*, *Dendroaspis angusticeps* from TZA, *Hemachatus haemachatus* from South Africa, *Echis romani* from NGA, *N. subfulva* from Uganda and *Naja haje* from Uganda were collected at the Liverpool School of Tropical Medicine and used in this study. Lyophilized snake venoms were solubilized in PBS and filter-sterilized prior to testing.

### Fractionation of whole venom

The proteins within *N. subfulva* and *N. mossambica* venom were separated using cation-exchange chromatography using an AKTA Pure M LC system. Buffers were freshly prepared and filtered through a 0.2 µm membrane immediately prior to use. Whole venom (15 mg) was dissolved in 3 ml 50 mM sodium phosphate, pH 5.2, and, after centrifuging, was loaded onto a 4.7 ml HiScreen SP HP column (Cytiva). The separation was then carried out using a 15-column volume gradient of 0–0.55 M NaCl in 50 mM sodium phosphate, pH 5.2. The flow rate was 0.6 ml min^−1^, and 2.0 ml fractions were collected. Elution was monitored at 214 and 280 nm.

### Dialysis of whole venom and fractionated venom

Whole venom and fractionated whole venom were dialysed into PBS in 100 µl aliquots to ensure that all samples were in the sample buffer before testing, using a Slide-A-Lyzer Mini 3.5K molecular weight cut-off dialysis cups (Thermo Scientific, USA) overnight in 1 l 1x PBS (Sigma, UK) at 4 °C, with gentle stirring.

### Filter sterilization

All whole venoms and dialysed products were filter-sterilized through an Ultrafree-MC 0.22 µm hydrophilic polyvinylidene fluoride centrifugal filter unit (Millipore, USA) at 13,000 r.p.m. for 1 min prior to testing.

### SDS-PAGE

Dialysed protein fractions from *N. mossambica* and *N. subfulva* were separated by SDS-PAGE. Samples were combined with LDS-4X buffer (Expedon, USA) containing 5% 2-mercaptoethanol (Sigma, UK) at a ratio of 3.5 : 6.5 µl and heated for 4 min at 98 °C. Thirty microlitres of each sample were loaded on a 16% TEO-Tricine gel (Expedon, USA) and ran at 180 V for 40 min using a Mini-PROTEAN electrophoresis system (BioRad, UK). Each gel was stained with InstantBlue Coomassie stain (Expedon, USA) for at least 24 h before visualization using the Gel Doc EZ system (BioRad, USA).

### Antimicrobial susceptibility testing

All antimicrobial susceptibility testing was performed in CA-MHB in a flat-bottomed 96-well plate against *E. coli* and whole and dialysed venom against *K. pneumoniae*. Lyophilized whole venom was reconstituted in PBS. Purified proteins were adjusted to a concentration of 20 µg ml^−1^ in a total volume of 50 µl of CA-MHB. When testing whole venom, dialysed whole venom and dialysed venom fractions, 10 µl of each sample, PBS and 100 µg ml^−1^ stock concentration of ciprofloxacin were diluted in 40 µl of CA-MHB. Bacterial cultures were standardized in CA-MHB to an OD measured at 600 nm (OD_600_) of between 0.8 and 1, then further diluted 1 : 1000 in CA-MHB. To all venom and protein samples (final concentration of 10 µg ml^−1^), PBS, ciprofloxacin (final concentration of 10 µg ml^−1^) and 50 µl of CA-MHB (positive control for bacterial growth), 50 µl of diluted culture was added, as well as 100 µl CA-MHB-only control, and statically incubated for 24 h at 37 °C. Following incubation, OD_600_ was measured using a microplate reader (BMG Labtech, UK), and relative growth for each sample was assessed in comparison to OD_600_ of the PBS control. The exact bacterial concentration of each inoculum was enumerated by further diluting the inoculum 1 out of 1,000 in PBS, and 100 µl was plated onto LB agar and incubated at 37 °C for 18 h. Colonies were visually enumerated to ensure that the bacterial inoculum corresponded to ~2–8×10^5^ c.f.u. ml^−1^.

### Mass spectrometry

Protein band extraction and mass spectrometry were performed by the Centre for Proteome Research, University of Liverpool (Liverpool, UK).

### Statistical analysis

All statistical analyses were performed in R (v4.5.1) using the Kruskal–Wallis test followed by Dunn’s test with Benjamini–Hochberg adjustment.

## Results

### Antimicrobial activity of whole venom

**Fig. 1.**
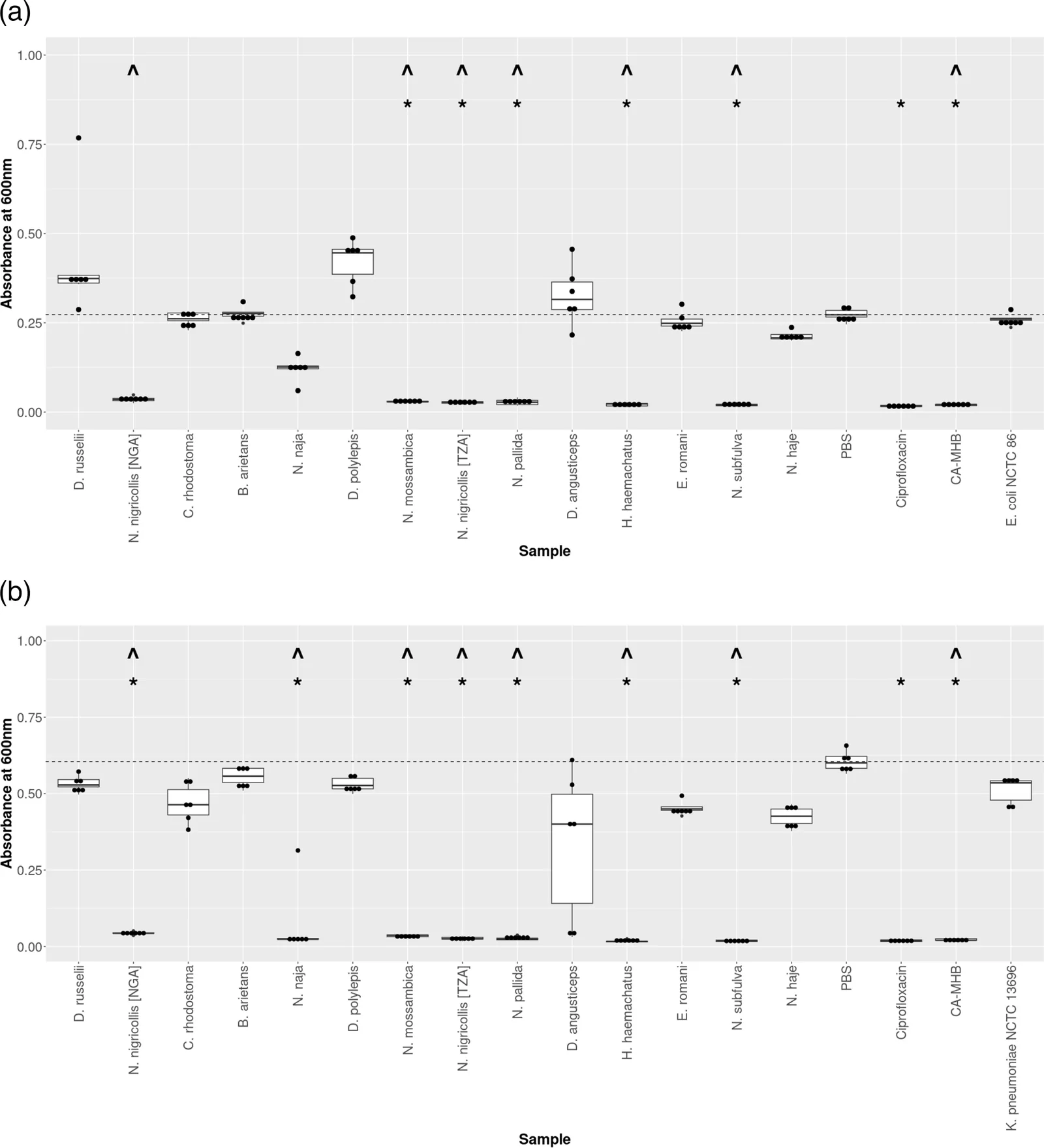
Effect of whole snake venom from *D. russelii*, *N. nigricollis* (NGA and TZA), *C. rhodostoma*, *B. arietans*, *N. naja*, *D*. *polylepis*, *N. mossambica*, *N. pallida*, *D. angusticeps*, *H. haemachatus*, *E. romani*, *N. subfulva* and *N. haje* in PBS on the growth of (a) *E. coli* NCTC 86 and (b) *K. pneumoniae* NCTC 13696 after 24 h. * indicates significant difference to PBS, while ^ indicates no significant difference to ciprofloxacin. Dashed line represents the mean OD_600_ of PBS.

We initially screened the antimicrobial activity of whole venom from 14 different snake species housed within the Centre for Snakebite Research and Interventions herpetarium at the Liverpool School of Tropical Medicine against a fully susceptible *E. coli* isolate. We found five whole venoms resulting in significant inhibition of growth (*P* values=0.0021–0.0336) and nine resulting in no significant change in bacterial growth compared to the PBS control (*P* values=0.063–0.989, Fig. 1). We used high concentrations of ciprofloxacin as a control to demonstrate complete inhibition of growth. From the 14 whole venom samples, we identified 6 whole snake venoms that resulted in near-complete inhibition of growth of *E. coli*, all of which were from the cobra genus *Naja*; *N. mossambica* (*P* value=0.221), *N. nigricollis* (TZA, *P* value=0.322), *N. nigricollis* (NGA, *P* value=0.126), *N. pallida* (*P* value=0.347), *H. haemachatus* (*P* value=0.665) and *N. subfulva* (*P* value=0.727, Fig. 1a).

These five species of cobra also demonstrated significant antimicrobial activity towards *K. pneumoniae*; *N. mossambica* (*P* value=0.0055), *N. nigricollis* (TZA, *P* value=0.00062), *N. nigricollis* (NGA, *P* value=0.0112), *N. pallida* (*P* value=0.00067), *H. haemachatus* (*P* value=0.00004) and *N. subfulva* (*P* value=0.00005, Fig. 1b) all demonstrated antimicrobial activity towards *K. pneumoniae* relative to PBS. The antimicrobial activity of all five caused near-complete inhibition of growth (*P* value=0.102–0.964). In addition, *N. naja* also demonstrated significant antimicrobial activity to *K. pneumoniae* relative to PBS (*P* value=0.00072) with no significant differences to 10 µg ml^−1^ ciprofloxacin (*P* value=0.457).

### Antimicrobial activity of dialysed whole venom

**Fig. 2.**
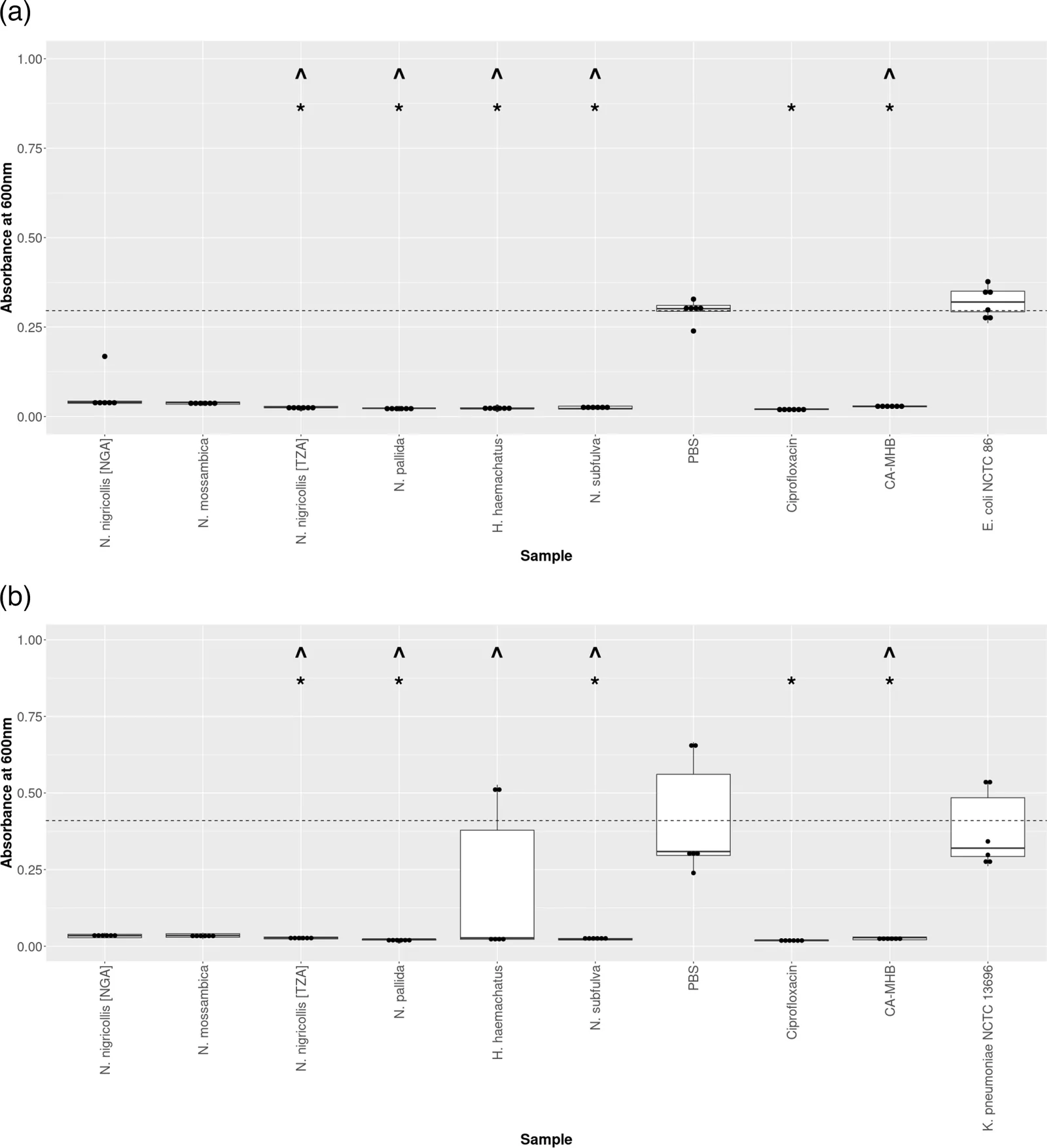
Effect of dialysed snake venom from (a) *N. nigricollis* (NGA), *N. mossambica*, *N. nigricollis* (TZA), *N. pallida*, *H. haemachatus* and *N. subfulva* in PBS on the growth of *E. coli* NCTC 86 after 24 h and (b) on the growth of *K. pneumoniae* NCTC 13696 after 24 h. * indicates significant difference to PBS, while ^ indicates no significant difference to ciprofloxacin. Dashed line represents the mean OD_600_ of PBS.

As snake venom is comprised of small-molecule components, as well as multiple protein classes [37], we dialysed six whole venoms that showed significant inhibition of growth to remove low-molecular-weight components below 3.5 kDa. We found that all the dialysed snake venoms tested retained their antimicrobial activity towards *E. coli* following dialysis, although only *H. haemachatus* (*P* value=0.0014), *N. subfulva* (*P* value=0.0030), *N. pallida* (*P* value=0.0016) and *N. nigricollis* (TZA, *P* value=0.0085) were significant compared to PBS, suggesting that the antimicrobial activity observed was due to the high-molecular-weight components above 3.5 kDa present in the venom (Fig. 2a). The inhibitory activity of four of the six dialysed whole venoms *N. nigricollis* (TZA, *P* value=0.252), *N. pallida* (*P* value=0.507), *H. haemachatus* (*P* value=0.558) and *N. subfulva* (*P* value=0.383) were not significantly different from the complete inhibition of growth by 10 µg ml^−1^ ciprofloxacin (Fig. 2a).

In contrast, only five of the dialysed venom retained activity towards *K. pneumoniae*, with only *N. nigricollis* (TZA, *P* value 0.032), *N. pallida* (*P* value=0.0020) and *N. subfulva* (*P* value=0.0084) significantly inhibiting growth compared to PBS (Fig. 2b). Additionally, they demonstrated near-complete inhibition of growth (*P* values=0.175–0.653). Both *N. pallida* and *H. haemachatus* demonstrated variable activity towards *K. pneumoniae* after dialysis which was assay dependent (Fig. 2b).

### Complexity of venom protein fractions

Due to the availability of a sufficient amount of material, we selected *N. mossambica* and *N. subfulva* whole venom to fractionate protein classes. While whole and dialysed venom from *N. mossambica* did not demonstrate statistically significant activity compared to PBS, there was clear and repeatable inhibitory activity towards both *E. coli* and *K. pneumoniae* to justify further exploration. The whole venom of *N. mossambica* and *N. subfulva* was separated using cation-exchange liquid chromatography, and each fraction was dialysed into PBS and filter-sterilized. We initially assessed the composition of the protein fractions using SDS-PAGE to determine whether each fraction contained one or multiple proteins prior to assessing the antimicrobial of the protein fractions. We found that there were no visible proteins in fractions 5–16 and 26–29 of *N. mossambica*, while fractions 17–25 and 30–35 contained only one or two visible proteins (Fig. S1A-D, available in the online Supplementary Material). In contrast, the composition of the protein fractions of *N. subfulva* was more complex, where fractions 5–10, 12, 17, 21–25, 28, 33–35 and 37–38 contained no visible protein bands and all other fractions contained between one and three proteins (Fig. S2A-D).

### Antimicrobial activity of protein fractions and protein identification

**Fig. 3.**
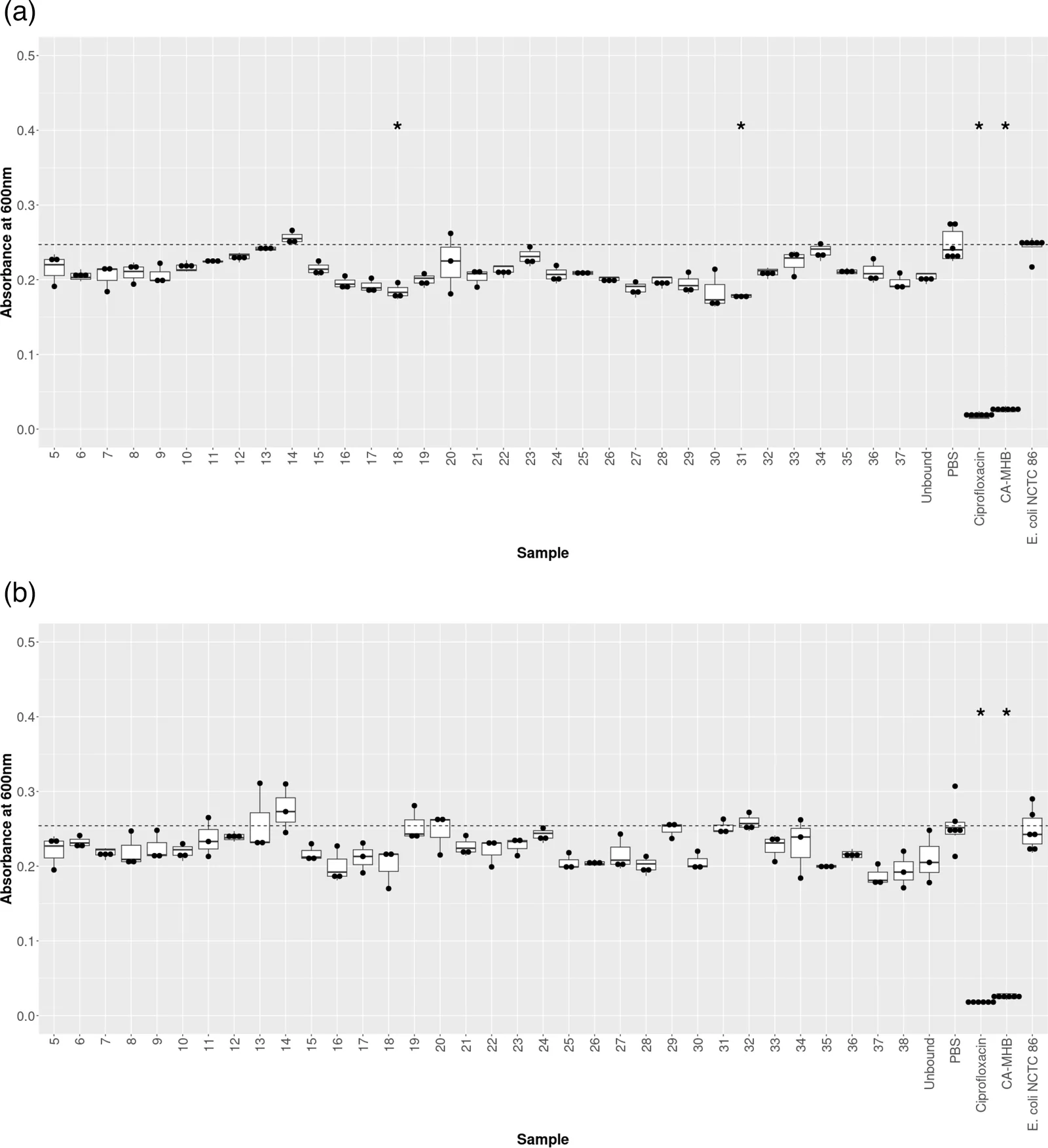
Effect of snake venom from (a) *N. mossambica* and (b) *N. subfulva* fractionated by cation-exchange liquid chromatography in PBS on the growth of *E. coli* NCTC 86 after 24 h. * indicates significant difference to PBS, while ^ indicates no significant difference to ciprofloxacin. Dashed line represents the mean OD_600_ of PBS.

While antimicrobial activity in the protein fractions did not cause complete inhibition of growth, such as to the degree observed in the corresponding whole snake venom, two fractions for *N. mossambica*, fraction 18 (*P* value=0.0364) and fraction 31 (*P* value=0.0166), significantly inhibited the growth of *E. coli* (Fig. 3a) compared to PBS. This suggests that the combined effect of the components of snake venom with antimicrobial activity results in the overall activity seen in the whole snake venom for each species. The top band of fraction 18 and the single band in the adjacent fraction 17 were identified as an acidic PLA_2_ with 83 and 61% coverage, respectively. The single band in both fraction 30 and fraction 31 was identified as a basic PLA_2_ with 92 and 78% coverage, respectively (Table 1).

**Table 1.** Protein fractions from *N. mossambica* and *N. subfulva* with activity towards *E. coli* NCTC 86, identified using mass spectrometry and the average mean OD_600_ relative to PBS

Snake species	Fraction	*P* value	Mass spectrometry top hit	Predicted percentage coverage	Relative average OD_600_
*N. mossambica*	17	0.0583	Acidic phospholipase	61%	0.78
	18 (top band)	0.0364	Acidic phospholipase	83%	0.75
	30	0.066	Basic phospholipase	92%	0.75
	31	0.0166	Basic phospholipase	78%	0.72
*N. subfulva*	18	0.2159	Long neurotoxin	75%	0.79
	26	0.1784	Short neurotoxin	37%	0.80

While no fractions from *N. subfulva* resulted in a statistically significant inhibition of *E. coli* realtive to PBS, we selected two fractions with single bands and relatively low OD_600_ compared to PBS. Fraction 18 (0.79 relative OD_600_) and fraction 26 (0.80 relative OD_600_) were identified by mass spectrometry as the 3FTxs long neurotoxin (75% coverage) and short neurotoxin (37% coverage), respectively (Table 1). Therefore, we have been able to identify the major protein class present in the protein fractions with significant antimicrobial activity, as well as protein classes with more subtle inhibitory effects on *E. coli*.

## Discussion

The development of novel antimicrobials is essential to combat the rising incidence of antimicrobial-resistant infections, and early-stage drug discovery projects to identify potential candidates are required. Therefore, there is a need to extensively examine underexploited resources for any antimicrobial properties to facilitate this, including snake venom. In this study, we have used a simple, iterative method to assess the antimicrobial activity of snake venom and identify the protein classes present in venom fractions with significant antimicrobial activity to enable potential further purification and drug development.

The first step of this method was the high-throughput assessment of the antimicrobial activity of whole snake venom towards a fully antimicrobial-susceptible isolate of *E. coli* and *K. pneumoniae*. As the venom of snakes is known to contain a microbiome [38, 39], the whole venom was filter-sterilized to ensure that the antimicrobial effect towards *E. coli* and *K. pneumoniae* was not masked by the growth of the constituents of the microbiome. Here, we were able to identify five whole venoms from the *Elapidae* family, four of which belonged to the *Naja* genus, which display antimicrobial activity towards *E. coli*, and seven which significantly inhibited *K. pneumoniae* from the *Elapidae* family, six of which belonged to the *Naja* genus. A recent study on the antimicrobial activity of snake venom identified multiple venoms from the *Viperinae* and *Elapidae* families demonstrating antimicrobial activity towards *E. coli*, *Bacillus subtilis*, *Pseudomonas aeruginosa* and *Staphylococcus aureus* [9].

Snake venom is known to contain a mixture of a variety of small molecules aside from the major protein classes [37]. To determine whether the antimicrobial activity observed in the whole venom assay was due to either low-molecular-weight components below 3.5 kDa or high-molecular-weight components above 3.5 kDa, we dialysed each venom in PBS to remove the low-molecular-weight components. We found that in all cases, the antimicrobial activity towards *E. coli* was retained post-dialysis, although the antimicrobial activity of only four of six was significantly different from PBS and no significant difference to the complete inhibition of growth by 10 µg ml^−1^ ciprofloxacin, and therefore confirmed that antimicrobial activity was due to high-molecular-weight components in the venom. In contrast, only three dialysed venoms retained significant antimicrobial activity towards *K. pneumoniae*. However, this does not preclude that low-molecular-weight components have a degree of antimicrobial activity, and we advocate that these are still assessed in other future studies.

Crude venom of *N. mossambica* has previously been shown to have antimicrobial activity towards *P. aeruginosa* [9] and *Candida* spp. [40], while little is known about the antimicrobial potential of *N. subfulva* venom. Fractionation of the whole venom of *N. mossambica* and *N. subfulva* highlighted that the antimicrobial activity observed was due to the combined effect of the components present in the venom, as the antimicrobial activity of the individual fractions was less than in the respective whole venom. By separating the protein classes in each venom, we were able to identify the fractions which contained proteins with observable inhibition of growth of *E. coli*. However, unlike other studies which have used clinical microbiological methods to determine antimicrobial activity [9, 28], this assay was able to detect subtle, but significant, changes in inhibition of growth.

Using mass spectrometry to identify proteins in single SDS-PAGE gel bands present in protein fractions, we were able to identify two protein classes present in four protein fractions from *N. mossambica* which have previously been described to have antimicrobial activity towards *E. coli*: basic PLA_2_ [41] and acidic PLA_2_ [42]. The two proteins present in the protein fractions of *N. subfulva* identified by mass spectrometry were previously unknown to have antimicrobial activity and are known venom neurotoxins [43, 44]. This shows that the assay can identify both proteins with known antimicrobial activity, as well as proteins which are not known to have antimicrobial activity.

This study was only focused on exploring the antimicrobial activity of venom towards a single strain of two bacterial species not representative of circulating clinical isolates. We used OD_600_ measurements specifically to identify inhibition of growth. This approach may identify antimicrobial proteins which have more subtle effects on bacterial growth but could still be exploited to develop antimicrobials. Additionally, we did not confirm the activity of the individual proteins, via isolation and purification, identified during fractionation and mass spectrometry. However, this assay can be used in high-throughput screening of whole venom from a wide range of species of snake, and indeed any venomous animal, towards an extended panel of both Gram-positive and Gram-negative bacteria, which can then be subject to iterative testing if they show antimicrobial activity to identify the protein classes responsible for the antimicrobial activity. An additional step can be included to concentrate those protein fractions without corresponding protein bands in SDS-PAGE to identify any proteins in low abundance in venom, but which still cause antimicrobial activity. Identification of protein classes through mass spectrometry, in protein fractions containing both single and multiple protein classes, will enable further purification of the proteins of interest to confirm antimicrobial activity and identification of the mode of action to enable further drug and inhibitor development. It is important to acknowledge the possibility of off-target and cytotoxic effects towards mammalian cells. Many of the proteins and polypeptides in snake venom are known to have cytotoxic effects [45], and therefore, it is important to test any potential candidates for drug development for cytotoxicity. However, PLA_2_ has previously been shown to have limited cytotoxic effects on human keratinocytes, which may open up avenues for development towards topical antimicrobials [46].

In further exploration, we recommend using a c.f.u. assay alongside the turbidity measurements to assay the effect of venom on bacterial viability. We also advocate for the isolation and purification of proteins showing potential antimicrobial activity. Following this, minimum inhibitory concentration, minimum bactericidal concentration and time kill assays should be used to benchmark their activity against current antimicrobials. These assays should also be performed against a diverse bacterial panel, including multiple species and strains.

## Supplementary material

10.1099/acmi.0.001151.v3 Supplementary Material 1.

## Abbreviations

AMR: antimicrobial resistance
CA-MHB: cation-adjusted Mueller–Hinton broth
3FTxs: three-finger toxins
LB: Luria–Bertani
NGA: Nigeria
OD_600_: OD measured at 600 nm
PLA_2_s: phospholipase A_2_s
TZA: Tanzania
