# Supplementary material for: Screening the antimicrobial properties of snake venoms: an iterative fractionation approach
Source: Access Microbiol. 2026 Sep 2;8(9):001151.v3. doi: 10.1099/acmi.0.001151.v3 (PMC13555706; doi:10.1099/acmi.0.001151.v3)
Supplement: Supplementary file 1 [file acmi-8-01151-s001.pdf]

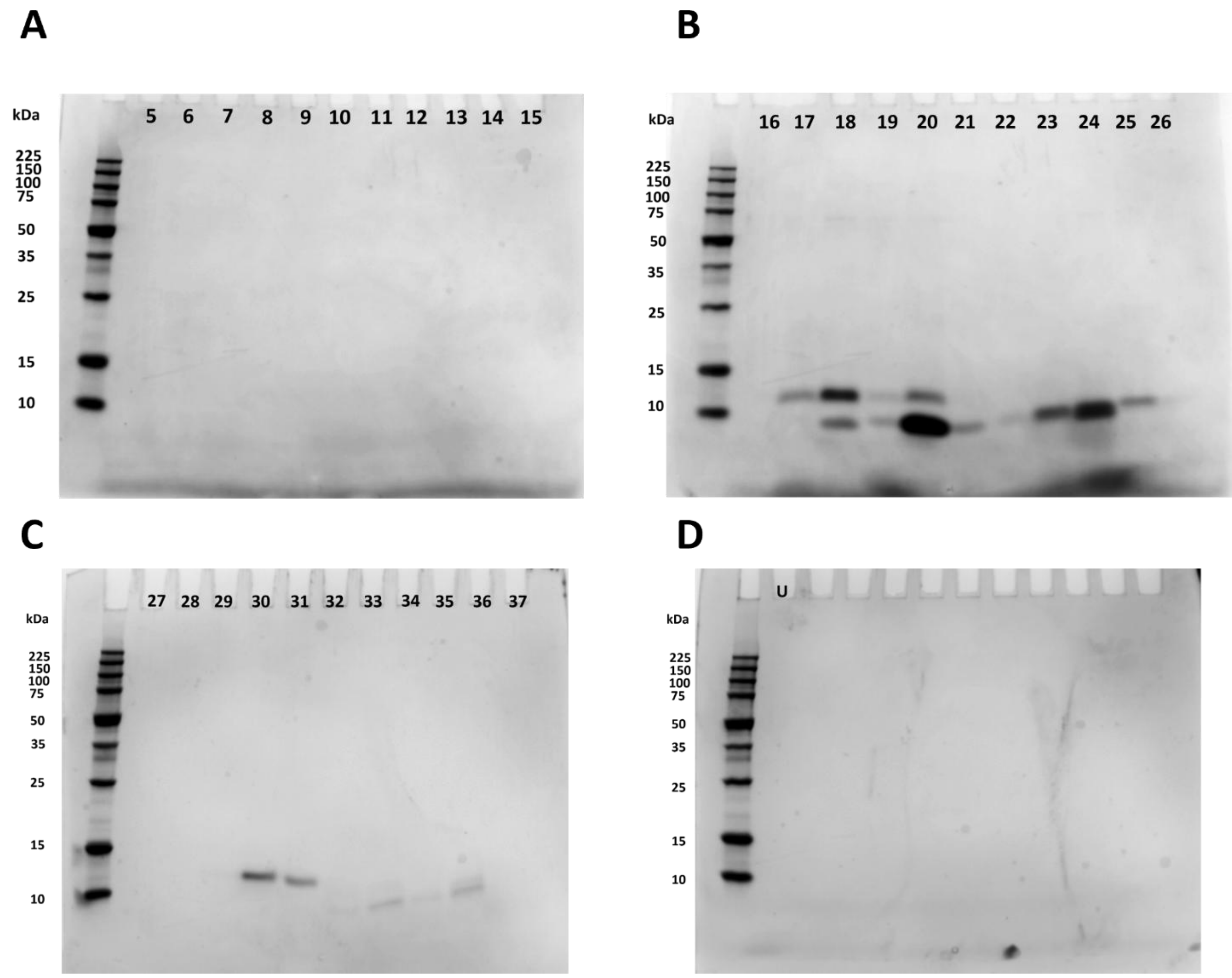

Figure S1: SDS page gels of *Naja mossambica* fractions, with A) fractions 5-15, B) fractions 16-26, C) fractions 27-37 and D) the unbound fraction.

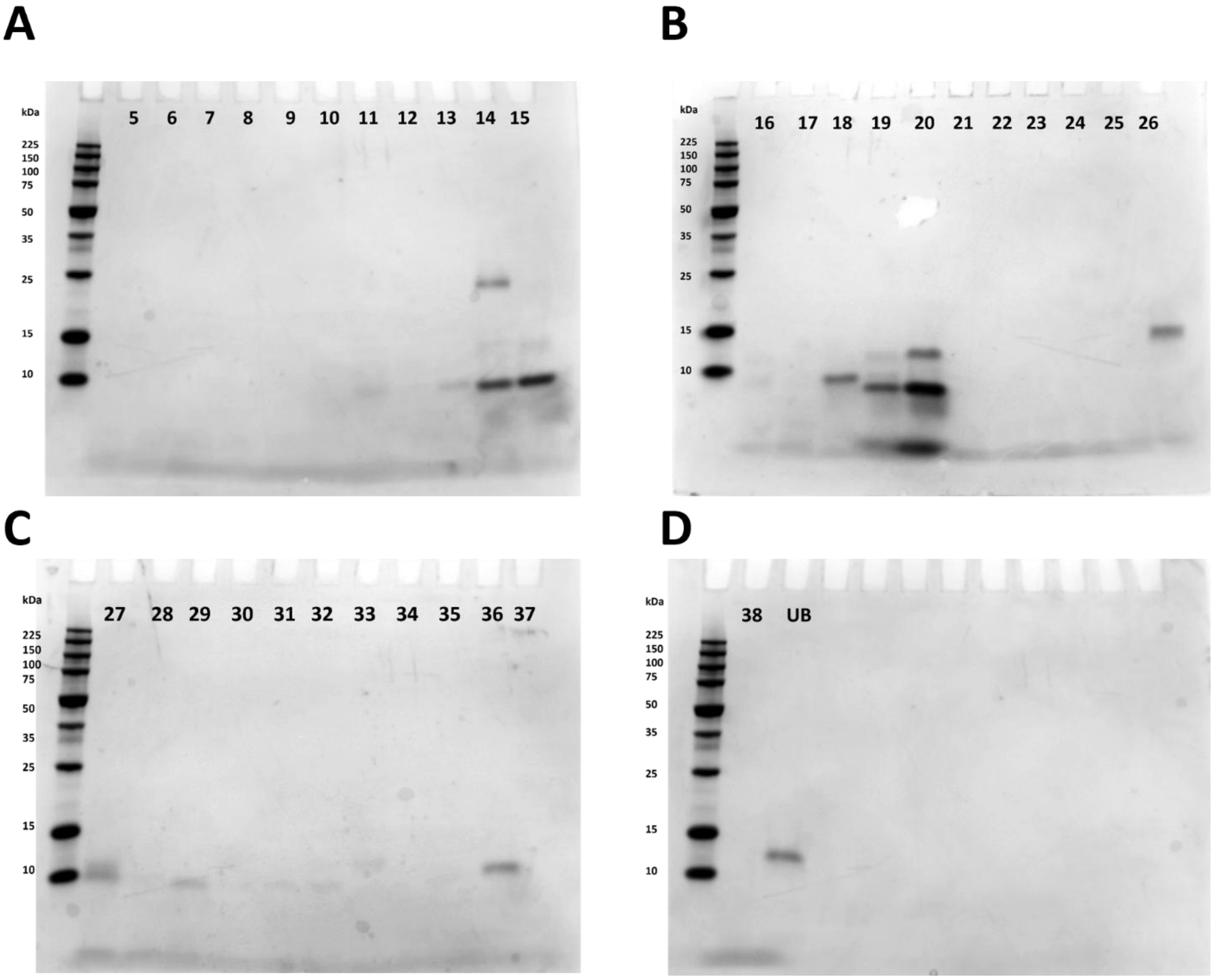

Figure S2: SDS page gels of *Naja subfulva* fractions, with A) fractions 5-15, B) fractions 16-26, C) fractions 27-37 and D) fraction 38 and the unbound fraction.
